# Proteome characterization of two contrasting soybean genotypes in response to different phosphorus treatments

**DOI:** 10.1093/aobpla/plab019

**Published:** 2021-04-14

**Authors:** Hongyu Zhao, Ahui Yang, Lingjian Kong, Futi Xie, Haiying Wang, Xue Ao

**Affiliations:** College of Agronomy, Shenyang Agricultural University, Shenyang 110866, China

**Keywords:** Differentially expressed proteins, phosphorus efficiency, proteomics, root, soybean

## Abstract

Phosphorus (P) is an essential element for the growth and development of plants. Soybean (*Glycine max*) is an important food crop that is grown worldwide. Soybean yield is significantly affected by P deficiency in the soil. To investigate the molecular factors that determine the response and tolerance at low-P in soybean, we conducted a comparative proteomics study of a genotype with low-P tolerance (Liaodou 13, L13) and a genotype with low-P sensitivity (Tiefeng 3, T3) in a paper culture experiment with three P treatments, i.e. P-free (0 mmol·L^−1^), low-P (0.05 mmol·L^−1^) and normal-P (0.5 mmol·L^−1^). A total of 4126 proteins were identified in roots of the two genotypes. Increased numbers of differentially expressed proteins (DEPs) were obtained from low-P to P-free conditions compared to the normal-P treatment. All DEPs obtained in L13 (660) were upregulated in response to P deficiency, while most DEPs detected in T3 (133) were downregulated under P deficiency. Important metabolic pathways such as oxidative phosphorylation, glutathione metabolism and carbon metabolism were suppressed in T3, which could have affected the survival of the plants in P-limited soil. In contrast, L13 increased the metabolic activity in the 2-oxocarboxylic acid metabolism, carbon metabolism, glycolysis, biosynthesis of amino acids, pentose phosphatase, oxidative phosphorylation, other types of *O*-glycan biosynthesis and riboflavin metabolic pathways in order to maintain normal plant growth under P deficiency. Three key proteins I1KW20 (prohibitins), I1K3U8 (alpha-amylase inhibitors) and C6SZ93 (alpha-amylase inhibitors) were suggested as potential biomarkers for screening soybean genotypes with low-P tolerance. Overall, this study provides new insights into the response and tolerance to P deficiency in soybean.

## Teaser

Soybean is an important food crop but its yield is affected by soil phosphorus (P) deficiency. We conducted a comparative proteomics study of two genotypes with contrasting responses to low-P. We observed that important metabolic pathways such as oxidative phosphorylation, glutathione metabolism and carbon metabolism were suppressed in the sensitive genotype. In contrast, the tolerant genotype increased the metabolic activity in pathways such as 2-oxocarboxylic acid metabolism, carbon metabolism, glycolysis, biosynthesis of amino acids, in order to maintain a normal growth under P deficiency. This study provides new insights into the response and tolerance to P deficiency in soybean.

## Introduction

Soybean (*Glycine max*) is a worldwide important economic and nutritional crop. Its seeds are enriched with high levels of proteins (40–50 %), fats (20–30 %) and vital phytochemicals, i.e. anthocyanins, tocopherols, isoflavones and saponins (Malenčić *et al.* 2012; Yamashita *et al.* 2020). Additionally, it plays cardinal ecological functions in cropping systems such as improving soil phosphorus (P) availability (Xia *et al.* 2013), soil carbon sequestration (Cong *et al.* 2015) and nitrogen fixation (Salvagiotti *et al.* 2008). It is a native crop of China and has more than 5000 years history (Lee *et al.* 2011; Sedivy *et al.* 2017). China is one of the main soybean producers in the world with a total production of 14 million tons in 2018 (Food and Agriculture Organization of the United Nations 2019). The Northeast area represents the main production areas in China, accounting for 44 % of total soybean production (Liu *et al.* 2008).

Phosphorus is the second most important macronutrient for plant, participating in various physiological and biochemical processes (Malhotra *et al.* 2018). Therefore, when P is limited in the soil, it restricts plant growth and productivity. Soil P deficiency is a worldwide problem restricting crop production (Smit *et al.* 2009; Balemi and Negisho 2012). Because of soil adsorption and fixation, P can be unavailable in soil, resulting in low utilization efficiency by plant. Phosphorus limitation is usually overcome by the application of P-containing fertilizers. However, excess use of P-containing fertilizers engenders environmental pollution (Lu 2003). Hence, it is crucial to enhance crop P-utilization efficiency in order to sustainably obtain stable and high productivity. Soybean production is significantly limited by low-P availability in soils (Yang *et al.* 2020). In China, the negative balance between production and consumption is compensated by importing yearly approximately up to 10 × 10^7^ tons of soybean (National Bureau of Statistics of China 2018). Developing low-P-tolerant soybean cultivars is one of the proposed strategies to close the gap between demand and supply.

Acquisition of P from soil is performed by the root; therefore, numerous studies have focused on root physiological and morphological traits under P-limited conditions. Plants with a fine root system characterized by high length, volume, biomass, specific root length are able to explore high soil volume thus can acquire more P (Wang *et al.* 2010). Also, it has been demonstrated that organic acids and phosphatase enzymes released from roots improve P availability and acquisition by plants (Dinkelaker *et al.* 1989; Hayes *et al.* 2000). Inter- and intraspecific variability for P-use efficiency has been reported in plants (Hammond *et al.* 2004; Kochian *et al.* 2004), which has facilitated the identification of numerous quantitative trait loci (QTLs) for P-use efficiency (Chen *et al.* 2009; Li *et al.* 2016; Yuan *et al.* 2017; Hacisalihoglu *et al.* 2018; Bernardino *et al.* 2019). However, most of the detected QTLs have very low contribution and heritability, making them unsuitable for breeding programs. Recently, *Omics* tools have been deployed to detect important molecular factors controlling P-use efficiency in plants and major players such as *OsPSTOL1*, *AVP1*, *PHO1*, *OsPHT1;6* and microRNA399 are being uncovered (Heuer *et al.* 2017).

Although studies have been carried out to investigate the molecular responses of soybean to the shortage of P in soil (Wang *et al.* 2010; Sha *et al.* 2016; O’Rourke *et al.* 2020; Yang *et al.* 2020), transcriptome and metabolome analyses were the focus of these studies. Proteins represent the actual functional molecules in the cell and are highly affected by abiotic stresses (Ahmad *et al.* 2016; Kosová *et al.* 2018). In soybean, few were focused on identifying key proteins involved in P-deficiency responses (Vengavasi *et al.* 2017). Importantly, comparing proteomes of genotypes with contrasting low-P tolerance levels will allow us to pinpoint major proteins and pathways involved in P-deficiency tolerance. In the present study, we explored the proteome differences in roots of low-P-tolerant and low-P-sensitive soybean genotypes under different concentrations of P using the Tandem Mass Tag (TMT)-based comparative proteomics approach.

## Materials and Methods

### Plant materials and growth conditions

Seeds of low-P-tolerant soybean genotype Liaodou 13 (L13) and low-P-sensitive genotype Tiefeng 3 (T3) previously studied by Yuxia *et al.* (2004) and Yang *et al.* (2020) were obtained from Shenyang Agricultural University. This research project began in 2004 with a screening of 220 soybean genotypes with different P efficiencies based on various P concentrations (Yuxia *et al.* 2004). From the test, 0.5 mM has been identified as the P treatment for a normal plant growth. Seeds were surface-sterilized with H_2_O_2_ and put in paper culture bags (8 * 16 cm, diameter * length). Five seeds were put in each bag in three groups for each genotype, i.e. P-free (0 mmol·L^−1^, P0), low-P (0.05 mmol·L^−1^, P1) and normal-P (0.5 mmol·L^−1^, P2). Seeds with normal-P served as control for comparative proteomics. The seed bags were placed in a light culture room, and the cultivation conditions were 16-h light/8-h dark, 18–28 °C temperature and 72-h light. At this stage, distilled water was used as culture medium. Upon emergence of cotyledonary leaf, seedlings were transferred to nutrient solution with three P levels: (0 mmol·L^−1^ KH_2_PO_4_ with KCl to maintain potassium concentration consistent with normal-P supply, P0), low-P (0.05 mmol·L^−1^ KH_2_PO_4_, P1) and normal-P (0.5 mmol·L^−1^ KH_2_PO_4_, P2). For other nutrients (compounds), concentrations were as follow: 4.5 mmol·L^−1^ KNO_3_, 1.2 mmol·L^−1^ NH_4_NO_3_, 3.6 mmol·L^−1^ CaSO_4_·2H_2_O, 0.25 mmol·L^−1^ MgSO_4_. Trace elements are: 9 μmol·L^−1^ H_3_BO_3_, 0.9 μmol·L^−1^ MnSO_4_, 0.9 μmol·L^−1^ ZnSO_4_, 1.5 μmol·L^−1^ CuSO_4_, 0.18 μmol·L^−1^ (NH_4_) 6Mo_7_O_24_ and 9 μmol·L^−1^ Fe-EDTA (pH adjusted to 5.8). Seedlings were supported on a 5-cm-thick styrofoam sheet at a spacing of 3 cm × 3 cm. The cotyledons were removed on third day of transfer to nutrient solution to minimize genotypic variation due to seed P content. Three replications with three seedlings each were maintained for all treatment combinations (Yang *et al.* 2020).

Nine days after the treatment, neat and consistent seedlings were selected for sampling in each treatment, and the average value of 3 plants per bag was taken as one repetition, and three repetitions were taken for each treatment. After taking out the whole plant, it was slowly washed with running water on the ground and root system of the plants was separated from the cotyledonary nodes. The roots were stored at −80 °C for proteomics study at: Chaya Biotech, Shanghai, China, following their standards procedures.

### Sample preparation

The roots were taken from −80 °C and quickly ground to powder in a mortar with liquid nitrogen. Hundred milligram of lyophilized powder was taken into a 1.5-mL centrifuge tube and we added 800 μL sodium dodecyl sulfate + dithiothreitol + Tris-HCl protein lysate (4% SDS, 100 mM Tris-HCl, 100 mM DL-Dithiothreitol, pH = 7.6). It was kept in boiling water bath at 100 °C for 5 min, followed by ultrasonification in ice bath for 10 min, again followed by 100 °C boiling water bath for 5 min. The tubes were centrifuged at 14 000 g for 30 min, and finally the supernatant was collected and filtered with 0.22-μm ultrafiltration tube.

### Protein quantification and sodium dodecyl sulfate–polyacrylamide gel electrophoresis

BiCinchoninic acid assay was used to optimize the concentration of proteins in each sample. Twenty microgram protein samples were used for sodium dodecyl sulfate–polyacrylamide gel electrophoresis (SDS-PAGE). The remaining samples were divided into 300-μg portions and stored in refrigerator at −80 °C. Finally the SDS-PAGE gel was scanned to obtain the gel map.

### Enzymatic hydrolysis

We took 300 μg of each sample for FASP enzymatic hydrolysis. Then, we added 200 μL of UA buffer (8 M Urea, 150 mM Tris–HCl, pH = 8.5) to the sample, mixed well, centrifuged at 14 000 g for 30 min at room temperature and discarded the filtrate. The same procedure was repeated two more times. We added 100 μL of IAA (50 mM IAA in UA), shook at 600 rpm for 1 min, incubated at 300 rpm for 30 min at room temperature, followed by centrifugation at 14 000 g for 30 min at room temperature. Next, 100 μL UA buffer was added, centrifuged at room temperature at 14 000 g for 30 min. The same procedure was repeated two more times. We added 100 μL of 25 mM·L^−1^ DS buffer (TEAB, pH = 8.5), centrifuged at 14 000 g for 30 min at room temperature. The same procedure was repeated two more times. Finally, the filtrate was discarded and 40 μL Trypsin buffer (6 μg Trypsin in 40 μL DS buffer) was added, and placed at constant temperature with gentle shaking (300 rpm, 18 h, 37 °C). Then, centrifuged at 14 000 g for 30 min at room temperature to collect the filtrate, replaced with a new collection tube, added 40 μL DS, centrifuged at 14 000 g at room temperature for 30 min, took the filtrate and quantified at abs (280 nm).

### Peptide labelling and gradation

We took 100 μg of each group of sample, according to instructions of the manufacturer: TMT6plex™ Isobaric Label Reagent Set (Thermo Scientific) instructions for labelling. The labelling scheme was set as TMT6 -126 TMT6 -127 TMT6 -128 TMT6 -129 TMT6 -130 TMT6 -131. All the labelled peptides were mixed separately and we used the high-pH (HpH) reversed-phase to pre-fractionate the peptides. Column: Gemini-NX 4.6 × 150 mm column (3 µm, 110 Å) (Phenomenex, 00F-4453-E0). Buffer: Buffer A was 10 mM ammonium acetate, pH = 10.0; Buffer B was 10 mM ammonium acetate, 90 % ACN, pH = 10.0. Instrument: 1100 Series HPLC Value System (Agilent). After HpH fractionation, we collected 40 fractions of the flow-through and elution for each set of markers, and combined them into 15 fractions according to the HpH chromatogram. We stored them at −80 °C. We combined the fractions according to their abundance from the chromatograph. Low abundance peptides were combined into one fraction and high abundance peptides were kept as a single fraction.

### Liquid chromatography–tandem mass spectrometry

Liquid phase A was 0.1 % formic acid in water, and liquid B was 0.1 % formic acid in acetonitrile (acetonitrile is 100 %). The analytical column (75 μm × 25 cm, 5 μm, 100 Å, C18) was equilibrated with 95 % A solution. The sample was loaded on the Thermo Scientific EASY trap column (100 μm × 2 cm, 5 μm, 100 Å, C18) by the autosampler, and then separated by chromatographic column. The relevant liquid phase gradient was as follows: 0–45 min, the linear gradient of liquid B was from 5 to 28 %; 45–80 min, the linear gradient of liquid B is from 28 to 90 %; 80–90 min, the liquid B was maintained at 90 %. The enzymolysis products were desalted and separated by capillary high-performance liquid chromatography and then analysed by Q Exactive mass spectrometer (Thermo Scientific). Analysis time: 90 min, detection method: positive ion, precursor scan range: 350–1800 m/z, first-level mass spectrometry resolution: 70 000, AGC target: 3e6, first-level maximum IT: 30 ms, number of scan ranges: 1, dynamic exclusion: 60.0 s. The mass-to-charge ratio of peptides and peptide fragments was collected as follows: 20 fragment maps (MS2 scan) are collected after each full scan, MS2 activation type: HCD, isolation window: 1.6 m/z, secondary mass spectrometry resolution: 35 000, microscans: 1, level 2 maximum IT: 100 ms, AGC target: 2e5, normalized collision energy: 35 eV, underfill ratio: 0.1 %.

### Mass spectrometry database analysis

The raw data of mass spectrometry analysis were RAW file, and the built-in software SEQUEST Proteome Discoverer 2.1 (Thermo Scientific) was used for protein annotation and quantitative analysis. The parameters used in this search were as follows: no restrictions on protein molecular weight, two missed cleavage, trypsin digestion, monoisotopic mass values, UniprotKB *G. max* (soybean) protein database, carbamidomethylation of cysteine as fixed modification, oxidation of methionine, peptide mass tolerance of ±20 ppm, fragment mass tolerance of 0.1 Da and peptide charge +1, +2 and +3. For quadrupole time-of-flight (Q-TOF), peptide mass tolerance and fragment mass tolerance were 20 ppm and 0.05 Da, respectively. Proteome Discoverer 2.1 performed screening with false discovery rate (FDR) ≤ 0.01 based on peptide identification results and quantitative analysis based on peptide peak intensity values. The original protein quantification result was the median of the peptide quantification result and was corrected by the sum of the reported ion peak intensity values of all channels. The final quantitative results were then normalized by the ratio of each label to the average of all channel intensity values and the median of the ratio. Functional classification of the proteins was performed based on the Kyoto Encyclopaedia of Genes and Genomes (KEGG) database (https://www.genome.jp/kegg/). The KEGG enrichment pathway with Bonferroni-corrected *P-*value < 0.05 was considered significant by using the hypergeometric test.

### Statistical analysis

All data were expressed as the mean ± standard error of mean. Differences among means of paired of groups were assessed by two-tailed *t*-test function in R software version 4.0.2. Differentially expressed proteins (DEPs) were identified with fold changes >1.5 or <0.67 and *P* < 0.05 (Zhang *et al.* 2017; Yang *et al.* 2019; Ye *et al.* 2021). Principal component analysis (PCA) was performed using xlstat package version 2020.1.

## Results

### Overview of proteome profiling in two contrasting soybean genotypes

**Figure 1.**
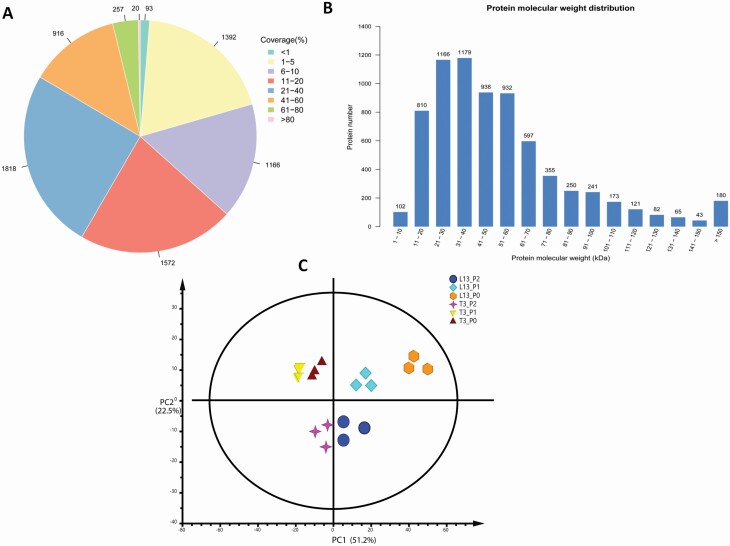
Evaluation of the proteome data. (A) Distribution of protein sequence coverage. (B) Protein mass distribution. (C) Principal component analysis based on protein expression data in the root samples of L13 and T3 subjected to three P levels (P0, P1 and P2).

The present study was performed to explore the variable expression of proteins in two genotypes of soybean under three P treatments: P-free (0 mmol·L^−1^, P0), low-P (0.05 mmol·L^−1^, P1) and normal-P (0.5 mmol·L^−1^, P2). L13 is a low-P-tolerant genotype while T3 is a low-P-sensitive genotype as demonstrated by Yuxia *et al.* (2004) and Yang *et al.* (2020). Seedlings grown under normal-P served as control for comparing the low-P and P-free treatments. We employed the TMT proteomics approach, which is an isotope-labelling method providing a relatively small error between groups, to profile the proteome of the different samples. A total of 41 678 peptides, 19 612 unique peptides and 4126 proteins were identified and quantified in a triplicate experiment **[see****Supporting Information—Table S1****]**. Sequence coverage of the majority of identified proteins was up to 60 % (Fig. 1A). Mass distribution of the identified proteins ranges from 5 to 568 kDa with majority of the proteins having molecular weights between 10 and 70 kDa (Fig. 1B). A PCA was performed based on protein quantification data across the 18 samples. As shown in Fig. 1C, all biological replicates were grouped together in the PCA, showing high degree of reproducibility. It also implies that proteome quantification was reliable. The PCA divided samples from T3 and L13 based on PC1 while P2 samples were distinct from P0 and P1 samples based on PC2. These results suggest that both genotypes have quite different response to P levels and P deficiencies greatly disturb the proteome in soybean.

### Differentially expressed proteins analysis in L13 genotype

**Figure 2.**
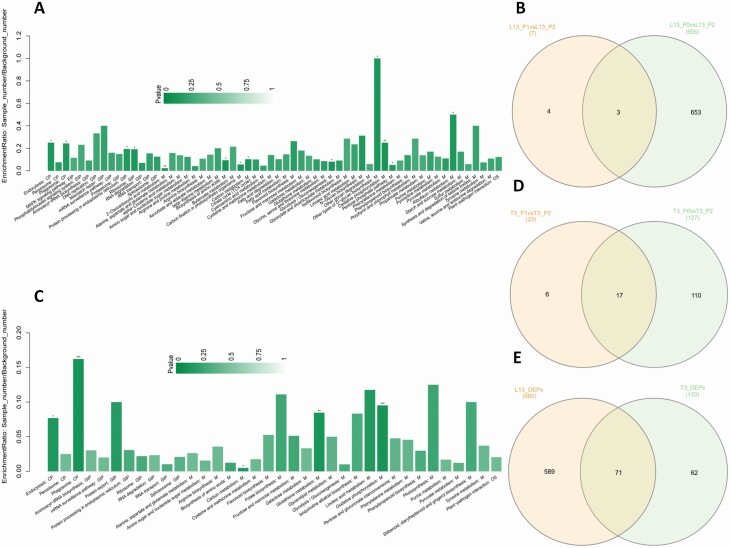
Analysis of the DEPs. (A) KEGG enrichment analysis of the DEPs between L13_P2 and L13_P0. (B) Venn diagram depicting the shared and unique DEPs between L13_P2 vs. L13_P1 and L13_P2 vs. L13_P0. (C) KEGG enrichment analysis of the DEPs between T3_P2 and T3_P0. (D) Venn diagram depicting the shared and unique DEPs between T3_P2 vs. T3_P1 and T3_P2 vs. T3_P0. (E) Venn diagram depicting the shared and unique DEPs between L13 and T3. *, ** or *** means corresponding KEGG pathways were significantly enriched at *P* < 0.05, 0.01 or 0.001 respectively.

In order to identify the DEPs between P treatments in L13, we compared L13_P2 (normal-P) to L13_P1 (low-P) and L13_P2 (normal-P) to L13_P0 (P-free). In L13_P2 vs. L13_P1, only seven DEPs were identified and all of them were upregulated in L13_P1 **[see****Supporting Information—Table S2****]**, indicating a positive response to low-P level in the soil. These DEPs were predicted to play various functions in response to low-P level **[see****Supporting Information—Table S2****]**. Concerning L13_P2 to L13_P0, a total of 656 DEPs were identified **[see****Supporting Information—Table S3****]**, showing that P0 induced a stronger response from soybean root proteome. All the DEPs were upregulated in L13_P0, implying that these proteins positively influence soybean response to P-free conditions. These DEPs were enriched in various KEGG pathways with the most enriched being 2-oxocarboxylic acid metabolism, carbon metabolism, biosynthesis of amino acids, glycolysis, oxidative phosphorylation, pentose phosphatase, other types of *O*-glycan biosynthesis and riboflavin metabolism in the metabolic pathways (Morcuende *et al.* 2007; Huang *et al.* 2008; Warren *et al.* 2011; Schlüter *et al.* 2013; Müller *et al.* 2015; Pant *et al.* 2015; Deng *et al.* 2018; Kc *et al.* 2018; Liu *et al.* 2020), likely to be crucial for tolerating P starvation in the soil (Fig. 2A). A cross-comparison of the two lists of DEPs showed that three core conserved proteins (I1KW20, I1K3U8 and C6SZ93) were constantly upregulated in L13 roots in response to low-P and P-free conditions (Fig. 2B; Table 1).

**Table 1.** Core conserved proteins responsive to P deficiency in L13 genotype.

Protein ID	Description	Fold change (L13_P1/L13_P2)	Fold change (L13_P0/L13_P2)	Regulation
I1KW20	Prohibitin	1.62	2.03	up/up
C6SZ93	AAI domain-containing protein	2.18	2.41	up/up
I1K3U8	AAI domain-containing protein	1.62	1.71	up/up

### Differentially expressed proteins analysis in T3 genotype

The pair-wise comparisons T3_P2 (normal-P) to T3_P1 (low-P) and T3_P2 (normal-P) to T3_P0 (P-free) were made in order to identify the DEPs involved in T3 response to P deficiencies in the soils. In T3_P2 vs. T3_P1, 23 DEPs were detected, including 22 downregulated and one upregulated proteins in T3_P1 **[see****Supporting Information—Table S4****]**. Out of these 23 DEPs, only four were annotated (lipoxygenase, 14-3-3-like protein, non-specific lipid-transfer protein, disease resistance protein/leucine-rich repeat (LRR) protein, non-specific lipid-transfer protein and urease), while the remaining DEPs were uncharacterized proteins. Concerning T3_P2 vs. T3_P0, 127 DEPs were identified, all being downregulated in T3_P0 **[see****Supporting Information—Table S5****]**. Majority of these DEPs are involved in oxidative phosphorylation, glutathione metabolism and carbon metabolism metabolic pathways (Fig. 2C). The higher number of DEPs induced by P0 compared to P1 in T3 further confirms that P starvation greatly affects the proteome in soybean root. In addition, the extensive downregulation of the DEPs in both low-P and P-free conditions in T3 illustrates that most of the metabolic processes and pathways are suppressed. A cross-comparison of the DEPs obtained from T3_P2 vs. T3_P1 and T3_P2 vs. T3_P0 revealed 17 core conserved proteins constantly suppressed by P deficiencies in T3 (Fig. 2D; Table 2).

**Table 2.** Core conserved proteins responsive to P deficiencies in T3 genotype.

Protein ID	Description	Isoforms (>90 % identity)	Fold change (T3_P1/T3_P2)	Fold change (T3_P0/T3_P2)	Regulation
K7LNG5	Uncharacterized protein	**Protein SRC1**	0.64	0.64	down/down
C6T1W3	Uncharacterized protein	Protein SRC1	0.62	0.59	down/down
B3TDK4	Lipoxygenase	—	0.61	0.52	down/down
Q9M5K7	14-3-3-like protein	—	0.66	0.46	down/down
I1K6M2	Uncharacterized protein	Protein P21	0.59	0.51	down/down
A0A0R0FEX5	Uncharacterized protein	**Protein SRC1**	0.38	0.49	down/down
A0A0R4J681	Uncharacterized protein	Glucose and ribitol dehydrogenase	0.55	0.34	down/down
I1LVB0	Uncharacterized protein	Miraculin protein	0.60	0.61	down/down
K7KJM5	Tyrosinase_Cu-bd domain-containing protein	—	0.54	0.66	down/down
I1M676	Uncharacterized protein	—	0.63	0.61	down/down
C6T3V8	Uncharacterized protein	Kunitz trypsin inhibitor 2	0.61	0.60	down/down
C6TFC1	Non-specific lipid-transfer protein	—	0.40	0.26	down/down
C6ZS00	Disease resistance protein/LRR protein-related protein	—	0.66	0.63	down/down
I1K3K3	Urease	—	0.63	0.40	down/down
A0A0R0J965	Uncharacterized protein	—	0.60	0.48	down/down
C6T488	Uncharacterized protein	Trypsin inhibitor A	0.49	0.42	down/down
C6SY13	Uncharacterized protein	Peroxisomal membrane protein PMP22	0.66	0.66	down/down

### Comparative analysis of DEPs between the two genotypes

Collectively, 660 and 133 unique DEPs were detected in L13 and T3 genotypes in response to P deficiencies, respectively. We compared DEPs of the two genotypes in order to identify common and genotype-specific DEPs. The result showed that 71 DEPs were commonly regulated in both genotypes under P deficiencies (Fig. 2E; **see****Supporting Information—Table S6**). However, all these shared DEPs were downregulated in T3 while upregulated in L13. This further underscores that the contrasting low-P tolerance observed between T3 and L13 genotypes is underpinned by their opposing proteome responses to P deficiencies. Furthermore, a high number of DEPs were specific to L13 and may contribute to its tolerance to limited P in the soil.

## Discussion

Yuxia *et al.* (2004) by screening a large population of soybean under P-limited conditions identified the genotypes L13 and T3 with contrasting tolerance levels. Recently, it has been shown that L13 is able to alter its root morphology in order to significantly improve P acquisition under P-limited conditions, a mechanism that T3 is unable to implement (Yang *et al.* 2020). In this study, we show that T3 tends to shut down the metabolism under P deficiencies conditions while L13 tends to boost the activity of proteins involved in key metabolic pathways. Such opposing behaviours could explain their contrasting tolerance to limited P in the soil.

Phosphorus is a principal component of various cellular molecules, such as ATP, nucleic acids, phospholipids, thus plays a crucial role in carbon metabolism and oxidative phosphorylation pathways (Huang *et al.* 2008). Carbon metabolism and oxidative phosphorylation pathways are known to generate energy and carbon essential for normal cellular function and for the synthesis of DNA, polyamines, amino acids, etc. (Nimir and Guisheng 2018; Meyer *et al.* 2019). Also, glutathione is a non-enzymatic antioxidant which enhances plant tolerance to different abiotic stresses such as salinity, drought and nutrient deficiency (Hasanuzzaman *et al.* 2011; Ramírez *et al.* 2013; Hasanuzzaman *et al.* 2017). Since P deficiency results in high accumulation of reactive oxygen species (ROS) in cells, glutathione plays cardinal role in scavenging excess ROS in P-limited environments (Juszczuk *et al.* 2001; Zhang *et al.* 2014). In this study, we observed that the low-P-sensitive genotype T3 limited the protein activity in the glutathione metabolism, carbon metabolism and oxidative phosphorylation pathways under P deficiencies conditions which ultimately will affect seedling normal growth and ROS scavenging ability, leading to low-P sensitivity.

In contrast, the low-P-tolerant genotype L13 stimulated the carbon metabolism and oxidative phosphorylation, in addition to glycolysis pathways which may have helped seedlings to keep normal growth and tolerate P stress. Besides, several other important pathways were boosted in L13 under P-stress conditions. For example, the biosynthesis of amino acids pathway representing the building blocks of proteins and fundamental for tissue repair, growth and nutrient absorption (Rai 2002) was upregulated under P-limited conditions. Similarly, proteins from the pentose phosphatase pathway (PPP) were upregulated. In PPP, the irreversible oxidative section occurring in non-photosynthetic cells such as in the root is a major source of the reducing equivalent NADPH for biosynthesis and maintaining the redox potential necessary to protect against oxidative stress (Dennis and Blakely 2000). Hence, by activating this pathway, L13 could control oxidative stress resulting from low-P levels in the soil (Valderrama *et al.* 2006). Similarly, riboflavin has been described as a potent antioxidant involved in various environmental stresses (Mittler 2002). This could explain the upregulation of the riboflavin metabolism pathway in L13 under P deficiencies conditions.

Identifying potential biomarkers for low-P tolerance is useful for a rapid screening of a large germplasm. In this study, we found three proteins (I1KW20 (prohibitins), I1K3U8 (alpha-amylase inhibitors) and C6SZ93 (alpha-amylase inhibitors)) constantly upregulated in L13 but not affected in T3 by low-P levels (Table 1). Both I1K3U8 and C6SZ93 proteins participate in the carbohydrate metabolism pathway which is essential for plant response to abiotic stress including low-P (Singh and Singh 1968; Rychter and Randall 1994; Rosa *et al.* 2009). I1KW20 contributes to mitochondrial biogenesis which is a specific pathway that supports photosynthetic processes and enables continuous survival during abiotic stress exposure in plants (Ahn *et al.* 2006; Welchen *et al.* 2014; Taylor 2018). Upregulation of proteins in these pathways as observed in L13 may have favoured the low-P tolerance.

These three proteins could be tested in a large germplasm to confirm their capacity to discriminate low-P-tolerant and -sensitive soybean genotypes. Such biomarkers could facilitate and accelerate breeding efforts towards low-P tolerance in soybean. An interesting follow-up experiment may be to test T3 mutant plants over-expressing the three putative key adaptive proteins in P-limited conditions. Besides, we found homologues of these proteins in several other plant species (I1KW20: A0A151UI57 (*Cajanus cajan*), V7BRY5 (*Phaseolus vulgaris*), A0A4D6M2C2 (*Vigna unguiculata*)/C6SZ93: A0A445DA28 (*Arachis hypogaea*), A0A1S3TVI8 (*Vigna radiata* var. *radiata*), Q43681 (*V. unguiculata*)/I1K3U8: A0A1S3UMP3 (*V. radiata* var. *radiata*), A0A151QZQ6 (*C. cajan*), V7CR23 (*P. vulgaris*), A0A1J7HDZ3 (*Lupinus angustifolius*)); hence, we predict that this work could be translated to other botanical species.

## Supporting Information

The following additional information is available in the online version of this article—

**Table S1.** List and characteristics of the proteins detected in root samples of two contrasting soybean genotypes (T3 and L13) subjected to three P treatments (P0, P1 and P2).

**Table S2.** Differentially expressed proteins between low-P (P1) and normal-P (P2) treatments in L13.

**Table S3.** Differentially expressed proteins between P-free (P0) and normal-P (P2) treatments in L13.

**Table S4.** Differentially expressed proteins between low-P (P1) and normal-P (P2) treatments in T3.

Table S5. Differentially expressed proteins between P-free (P0) and normal-P (P2) treatments in T3.

Table S6. Differentially expressed proteins commonly regulated in both genotypes under P deficiencies.

plab019_suppl_Supplementary_Materials

## Data Availability

All data used in this study are available within the manuscript and its supplementary files.

## Notes

**Phenome, Genome & Environment. Chief Editor:** Colleen Doherty
